# Supplementary figures and images for: Association between environmental and climatic risk factors and the spatial distribution of cystic and alveolar echinococcosis in Kyrgyzstan
Source: PLoS Negl Trop Dis. 2021 Jun 23;15(6):e0009498. doi: 10.1371/journal.pntd.0009498 (PMC8259979; doi:10.1371/journal.pntd.0009498)

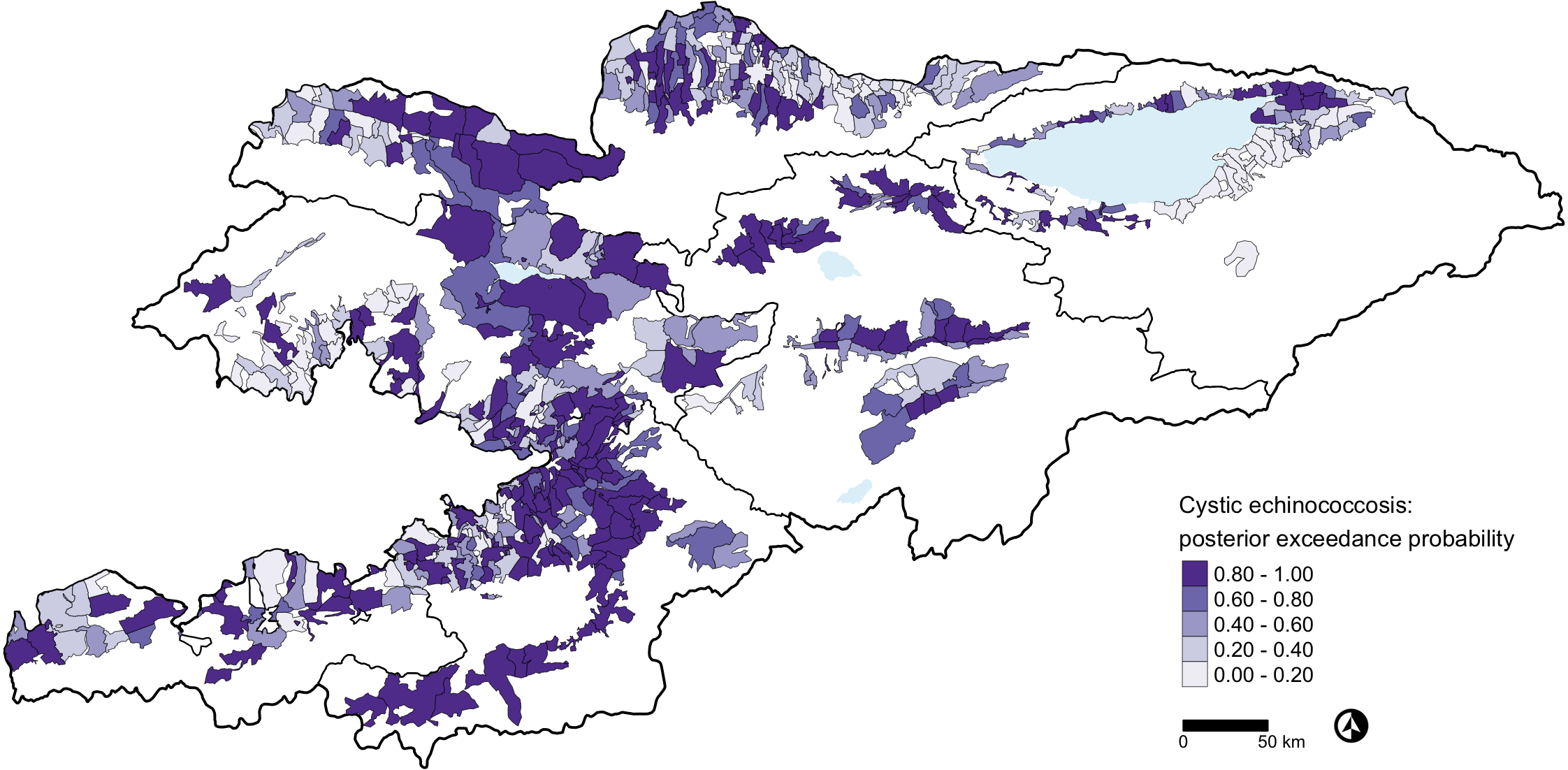

Supplement: S1 Fig — The PEP ranges between 0 and 1 and provides evidence of significant excess of CE or AE risk within individual areas. We used the third level administrative boundaries of Kyrgyzstan as provided by REACH, a joint initiative of IMPACT, ACTED, and the UN Operational Satellite Applications Programme (UNOSAT) under a humanitarian license. The shapefiles were subsequently edited to add missing polygons. The code and files for the map are available at: https://git.math.uzh.ch/reinhard.furrer/Echin_kgz. Shape files for third level administrative regions are now freely available from the United Nations Office for the Coordination of Humanitarian Affairs https://data.humdata.org/dataset/kyrgyzstan-administrative-boundaries. (TIF) [file pntd.0009498.s001.tif]

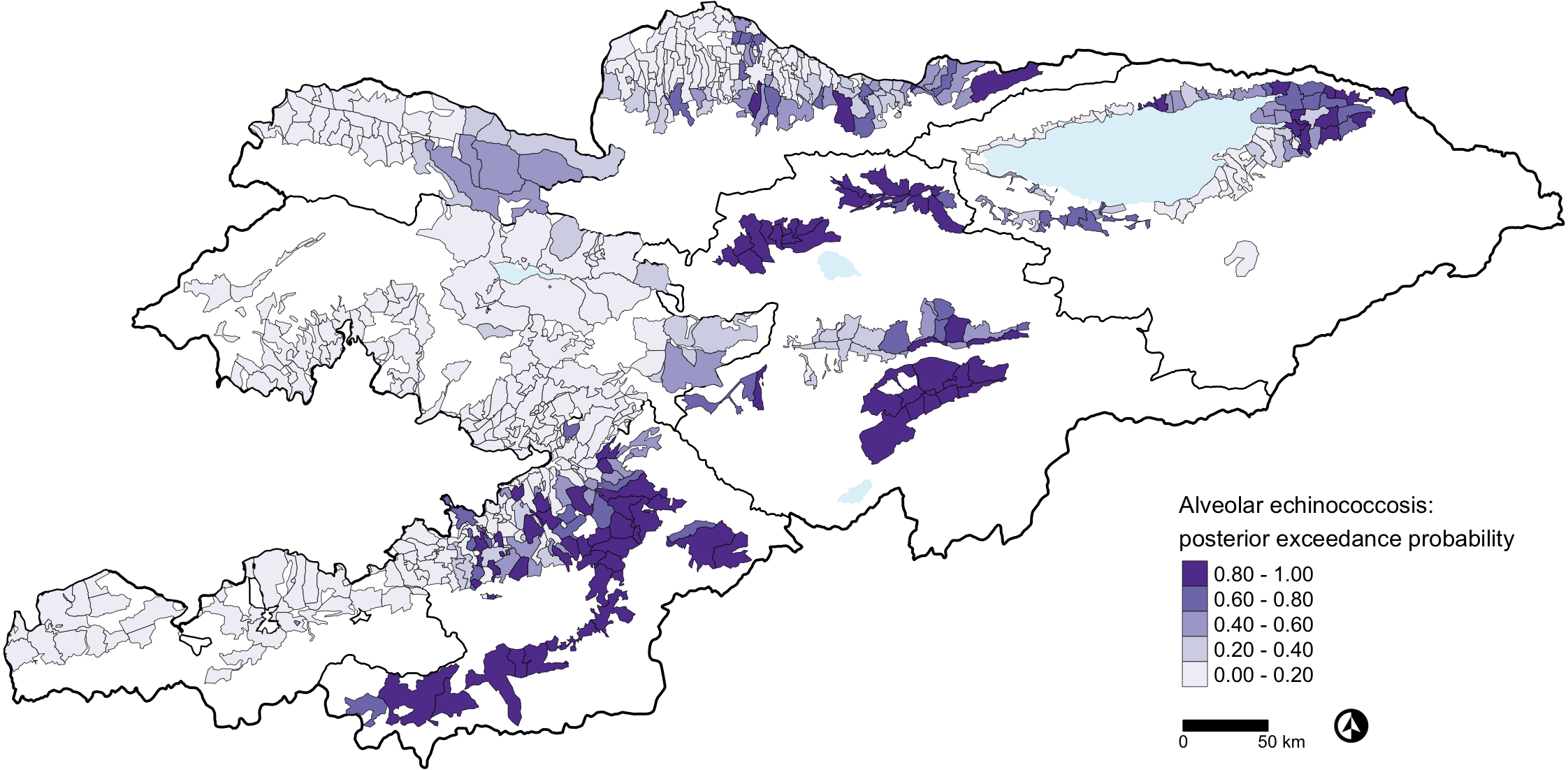

Supplement: S2 Fig — The PEP ranges between 0 and 1 and provides evidence of significant excess of CE or AE risk within individual areas. We used the third level administrative boundaries of Kyrgyzstan as provided by REACH, a joint initiative of IMPACT, ACTED, and the UN Operational Satellite Applications Programme (UNOSAT) under a humanitarian license. The shapefiles were subsequently edited to add missing polygons. The code and files for the map are available at: https://git.math.uzh.ch/reinhard.furrer/Echin_kgz. Shape files for third level administrative regions are now freely available from the United Nations Office for the Coordination of Humanitarian Affairs https://data.humdata.org/dataset/kyrgyzstan-administrative-boundaries. (TIF) [file pntd.0009498.s002.tif]
