# Supplementary material for: Association between environmental and climatic risk factors and the spatial distribution of cystic and alveolar echinococcosis in Kyrgyzstan
Source: PLoS Negl Trop Dis. 2021 Jun 23;15(6):e0009498. doi: 10.1371/journal.pntd.0009498 (PMC8259979; doi:10.1371/journal.pntd.0009498)
Supplement: S1 Table — (DOC) [file pntd.0009498.s003.doc]

**S1 Table.** **Geospatial variables (no. 190) on potential environmental and climatic risk factors for cystic echinococcosis and alveolar echinococcosis in Kyrgyzstan collected for this analysis**.

| **Variable name** | **Description** | **Format** | **Unit** | **Spatial resolution** | **Link** |
| --- | --- | --- | --- | --- | --- |
| Seasonal and annual surface air temperature | Seasonal^a^ (Winter, spring, summer, autumn) 2000, 2005, 2010 (no. 12)  Annual 2000, 2005, 2010 (no. 3) | Raster | °C | 0.5 x 0.625° (~ 50 km) | <https://giovanni.gsfc.nasa.gov/giovanni/>  dataset: surface air temperature (M2TMNXFLX v5.12.4) |
| Seasonal and annual precipitation | Seasonal^a^ (Winter, spring, summer, autumn) 2000, 2005, 2010 (no. 12)  Annual 2000, 2005, 2010 (no. 3) | Raster | mm h^-1^ | 0.25° (~ 25 km) | <https://giovanni.gsfc.nasa.gov/giovanni/>  dataset: Multisatellite Precipitation. Precipitation Rate (TRMM_3B43 v7) |
| Seasonal and annual soil moisture | Seasonal^a^ (Winter, spring, summer, autumn) 2000, 2005, 2010 (no. 12)  Annual 2000, 2005, 2010 (no. 3) | Raster | kg of water m^-3^ | 0.5 x 0.625° (~ 50 km) | <https://giovanni.gsfc.nasa.gov/giovanni/>  dataset: soil water profile (M2TMNXLND v5.12.4) |
| Seasonal and annual NDVI^b^ | Seasonal^a^ (Winter, spring, summer, autumn) 2000, 2005, 2010 (no. 12)  Annual 2000, 2005, 2010 (no. 3) | Raster | NDVI | 0.05° (~ 5 km) | <https://giovanni.gsfc.nasa.gov/giovanni/>  dataset: moderate-resolution imaging spectroradiometer MODIS-Terra MOD13C2 v006. |
| Seasonal and annual EVI^c^ | Seasonal^a^ (Winter, spring, summer, autumn) 2000, 2005, 2010 (no. 12)  Annual 2000, 2005, 2010 (no. 3) | Raster | EVI | 0.05° (~ 5 km) | <https://giovanni.gsfc.nasa.gov/giovanni/>  dataset: moderate-resolution imaging spectroradiometer MODIS-Terra MOD13C2 v006. |
| Seasonal and annual Land Surface Temperature | Seasonal^a^ (Winter, spring, summer, autumn) 2000, 2005, 2010 (no. 12)  Annual 2000, 2005, 2010 (no. 3) | Raster | °C | 0.5 x 0.625° (~ 50 km) | <https://giovanni.gsfc.nasa.gov/giovanni/>  dataset: MERRA Model, MATMNXSLV v5.2.0. (*16*) |
| Monthly precipitation | Monthly (January-December), 1970-2000 (no. 12) | Raster | mm | 30" (~ 1 km) | <http://worldclim.org/version2> |
| Monthly minimum, maximum, and average temperature | Monthly (January-December), 1970-2000 (no. 36) | Raster | °C | 30" (~ 1 km) |
| Monthly solar radiation | Monthly (January-December), 1970-2000 (no. 12) | Raster | kJ m^-2^ day^-1^ | 30" (~ 1 km) |
| Distance to GHSL ESA CCI LC^d^   - cultivated area edges - woody-tree area edges - herbaceous area edges - sparse vegetation area edges - aquatic vegetation area edges - shrub area edges - bare area edges - artificial surface (urban) edges - built-settlement area edges | Annual 2000, 2005, 2010 (no. 27)  The values of the raster are the distances in km from the cell canters to the nearest feature. | Raster | km | 3” (~ 0.1 km) | <https://dx.doi.org/10.5258/SOTON/WP00644>  <https://www.worldpop.org/project/categories?id=14> |
| - OSM^e^ major roads | Annual, 2016 (no. 1) | Raster | km | 3” (~ 0.1 km) |
| - OSM major waterways | Annual, 2016 (no. 1) | Raster | km | 3” (~ 0.1 km) |
| - inland waterbodies | Average 2000-2012 (no. 1) | Raster | km | 3” (~ 0.1 km) |
| Night-time lights VRIIS^f^ | Annual 2000, 2005, 2010 (no=3) | Raster | nanoWatts cm^-2^ sr^-1^ | 3” (~ 0.1 km) |
| Elevation | 2000 (no. 1) | Raster | m | 3” (~ 0.1 km) |
| Slope | 2000 (no. 1) | Raster | Degree | 3” (~ 0.1 km) |
| Population density | Annual 2000, 2005, 2010 (no. 3)  Ratio between population and square km per community | Csv file | Population km^-2^ | Community |
| Pasture area fraction | Annual, 2000 (no. 1) | Raster | Area fraction | 5' (~ 10 km) | <http://www.earthstat.org/cropland-pasture-area-2000/> |
| Cropland area fraction | Annual, 2000 (no. 1) | Raster | Area fraction | 5' (~ 10 km) |

^a^Winter: from December through February; spring: from March through May; summer: from June through August, autumn: from September through November.

^b^NDVI, Normalized Difference Vegetation Index.

^c^EVI, Enhanced vegetation index.

^d^GHSL, Global Human Settlement Layer; ESA: European Space Agency, CCI: Climate Change Initiative, LC: Land Cover

^e^OSM: Open Street Map

^f^VRIIS: Visible Infrared Imaging Radiometer Suite

Last access links: 13/08/2020
