# Supplementary material for: Association between environmental and climatic risk factors and the spatial distribution of cystic and alveolar echinococcosis in Kyrgyzstan
Source: PLoS Negl Trop Dis. 2021 Jun 23;15(6):e0009498. doi: 10.1371/journal.pntd.0009498 (PMC8259979; doi:10.1371/journal.pntd.0009498)
Supplement: S2 Table — (DOC) [file pntd.0009498.s004.doc]

**S2 Table.** **Variables (no. 13) with significant correlation (p<0.05) with mean annual temperature in 2005 in Kyrgyzstan.**

| **Variable name** | **Description** | **Correlation index with 2005 mean annual temperature** |
| --- | --- | --- |
| Seasonal surface air temperature | 2000 Winter mean temperature | 0.9925 |
| Seasonal surface air temperature | 2000 Summer mean temperature | 0.9956 |
| Seasonal surface air temperature | 2000 Spring mean temperature | 0.9993 |
| Seasonal surface air temperature | 2000 Autumn mean temperature | 0.9957 |
| Seasonal surface air temperature | 2005 Winter mean temperature | 0.9804 |
| Seasonal surface air temperature | 2005 Summer mean temperature | 0.9958 |
| Seasonal surface air temperature | 2005 Spring mean temperature | 0.9975 |
| Seasonal surface air temperature | 2005 Autumn mean temperature | 0.9995 |
| Seasonal surface air temperature | 2010 Winter mean temperature | 0.9902 |
| Seasonal surface air temperature | 2010 Summer mean temperature | 0.9969 |
| Seasonal surface air temperature | 2010 Spring mean temperature | 0.9961 |
| Seasonal surface air temperature | 2010 Autumn mean temperature | 0.9988 |
| Annual surface air temperature | 2000 annual mean temperature | 0.9995 |
