# Supplementary material for: Association between environmental and climatic risk factors and the spatial distribution of cystic and alveolar echinococcosis in Kyrgyzstan
Source: PLoS Negl Trop Dis. 2021 Jun 23;15(6):e0009498. doi: 10.1371/journal.pntd.0009498 (PMC8259979; doi:10.1371/journal.pntd.0009498)
Supplement: S4 Table — (DOC) [file pntd.0009498.s006.doc]

**S4 Table. Communities where no alveolar echinococcosis cases where reported in the study period that are at risk for alveolar echinococcosis (relative risk higher than 1) according to the Besag, York and Mollié model, Kyrgyzstan, 2014-2016.**

| **Region** | **District** | **Community** | **Community’s population** | **AE^a^ cases** | **Relative Risk** |
| --- | --- | --- | --- | --- | --- |
| Naryn | Jumgal | Kyzyl-Jyldyz | 1667 | 0 | 4.42 |
| Chui | Kemin | Bordu | 125 | 0 | 4.08 |
| Naryn | At-Bashy | Ak-Dj?r | 3208 | 0 | 2.99 |
| Naryn | Kochkor | Kosh-Dobo | 2843 | 0 | 2.61 |
| Naryn | Kochkor | Kok-Jar | 2308 | 0 | 2.49 |
| Naryn | At-Bashy | Kara-Suu | 5141 | 0 | 2.46 |
| Naryn | Ak-Tala | Jerge-??l? | 2298 | 0 | 2.43 |
| Osh | Uzgen | Kara-Tash | 2069 | 0 | 2.36 |
| Chui | Kemin | Ak-Tuz | 580 | 0 | 2.09 |
| Osh | Kara-Kulja | Oy-Tal | 3004 | 0 | 2.08 |
| Issyk-Kul | Tup | Ak-Bula | 1062 | 0 | 2.05 |
| Issyk-Kul | Ak-Suu | Ak-Chiy | 3162 | 0 | 1.97 |
| Naryn | Naryn | Dostuk | 750 | 0 | 1.85 |
| Osh | Kara-suu | Kyzyl-Suu | 4888 | 0 | 1.80 |
| Issyk-Kul | Ak-Suu | Boz-Uchuk | 6031 | 0 | 1.79 |
| Osh | Kara-Kulja | Karaguz | 6161 | 0 | 1.75 |
| Issyk-Kul | Tup | Karasaev | 3978 | 0 | 1.72 |
| Chui | Kemin | Chon-Kemin | 3788 | 0 | 1.70 |
| Chui | Kemin | Ilichev | 2198 | 0 | 1.52 |
| Chui | Alamudun | Arashan | 3961 | 0 | 1.50 |
| Naryn | Naryn | Chet-Nur | 5294 | 0 | 1.48 |
| Naryn | Naryn | Kazan-Kuigan | 1115 | 0 | 1.44 |
| Issyk-Kul | Issyk-Kul | Oruktu | 4068 | 0 | 1.43 |
| Chui | Kemin | Boroldoi | 1893 | 0 | 1.41 |
| Issyk-Kul | Issyk-Kul | Abdrahmanov | 2709 | 0 | 1.36 |
| Chui | Kemin | Kyzyl-Oktyabr | 3366 | 0 | 1.33 |
| Issyk-Kul | Tup | Yssyk-Kol | 2266 | 0 | 1.32 |
| Osh | Uzgen | Keldyk | 5357 | 0 | 1.32 |
| Chui | Alamudun | Baitik | 2541 | 0 | 1.32 |
| Osh | Uzgen | Altyn Bulak | 3328 | 0 | 1.32 |
| Osh | Kara-Kulja | Kenesh | 3742 | 0 | 1.32 |
| Issyk-Kul | Karakol (city) | Pristan-Prejeval | 2917 | 0 | 1.29 |
| Chui | Kemin | Kara-Bulak | 2235 | 0 | 1.28 |
| Issyk-Kul | Tup | Mihailov | 3841 | 0 | 1.27 |
| Issyk-Kul | Ak-Suu | Chelpek | 6898 | 0 | 1.27 |
| Naryn | Naryn | Jergetal | 5228 | 0 | 1.26 |
| Issyk-Kul | Issyk-Kul | Sadyr-Ake | 5324 | 0 | 1.18 |
| Chui | Ysyk-Ata | Yssyk-Ata | 5025 | 0 | 1.16 |
| Naryn | Ak-Tala | Terek | 1173 | 0 | 1.16 |
| Issyk-Kul | Tup | Toguz-Bulak | 1946 | 0 | 1.15 |
| Talas | Talas | Kara-Oi | 5252 | 0 | 1.13 |
| Talas | Talas | Kalby | 5454 | 0 | 1.11 |
| Chui | Chui | Sailyk | 2171 | 0 | 1.10 |
| Naryn | Naryn | Sary-Oy | 1113 | 0 | 1.10 |
| Osh | Uzgen | Salam-Alik | 7440 | 0 | 1.09 |
| Issyk-Kul | Ton | Kadjysay | 4222 | 0 | 1.07 |
| Naryn | Ak-Tala | Ak-Talaa | 1177 | 0 | 1.06 |
| Issyk-Kul | Ak-Suu | Karakol | 3006 | 0 | 1.04 |
| Issyk-Kul | Ak-Suu | Teplokliuch | 11010 | 0 | 1.04 |

^a^AE: alveolar echinococcosis
