# Supplementary material for: Association between environmental and climatic risk factors and the spatial distribution of cystic and alveolar echinococcosis in Kyrgyzstan
Source: PLoS Negl Trop Dis. 2021 Jun 23;15(6):e0009498. doi: 10.1371/journal.pntd.0009498 (PMC8259979; doi:10.1371/journal.pntd.0009498)
Supplement: Attachment — Submitted filename: pointToPoint_answers.docx [file pntd.0009498.s007.docx]

**Comments from PLOS NTDs Editor, Reviewers, and point-by-point replies from the Authors**

[Editor comments	1](#_Toc64816671)

[Reviewer's Responses to Questions	5](#_Toc64816672)

[Key Review Criteria Required for Acceptance?	5](#_Toc64816673)

[Results	8](#_Toc64816674)

[Conclusions	9](#_Toc64816675)

[Editorial and Data Presentation Modifications?	11](#_Toc64816676)

[Summary and General Comments	15](#_Toc64816677)

[Figure Files	18](#_Toc64816678)

[Data Requirements	18](#_Toc64816679)

# Editor comments

| **Comment #** | **Editor comment** | **Authors answer** |
| --- | --- | --- |
|  | Thank you very much for submitting your manuscript "Association between environmental and climatic risk factors and the spatial distribution of cystic and alveolar echinococcosis in Kyrgyzstan" for consideration at PLOS Neglected Tropical Diseases. As with all papers reviewed by the journal, your manuscript was reviewed by members of the editorial board and by several independent reviewers. In light of the reviews (below this email), we would like to invite the resubmission of a significantly-revised version that takes into account the reviewers' comments.  Please address all reviewer concerns in an updated manuscript. Also, take note to cite all sources using PLOS NTD approved citation styles (including websites and new media). | We thank the reviewers and the Editors for the constructive feedback. We have addressed all the points and concerns from the reviewers as indicated below. Their comments and suggestions were very helpful to improve the manuscript. |
| C 1 | In addition to addressing concerns raised by the reviewers, please consider to expand your explanation in the methods and table S1 of which variables were excluded due to collinearity - and try to explain for each excluded variable: excluded due to collinearity with which retained variables?  In particular, for the significant predictor for AE of 10-year lagged temperature, were there other variables that were excluded that were highly collinear with 10-year lagged temperature? If so, consider to be explicit about those and add speculation in the discussion as to the potential mechanisms involving the other excluded variables that were also collinear with 10-year lagged temperature (if any). | We thank the Editor for this very useful suggestion. Given the relatively long latency period of AE and CE, we assessed the explanatory variables across different time periods for time-variable events such as temperature and rainfall. Multicollinearity was thus expected when variables computed in different time points would display limited temporal variations. Among the 190 variables we considered initially (S1 Table), we observed significant correlation (⍴ >0.9, p<.05) between the variable *mean 10-year lag temperature* (namely, the mean annual temperature in 2005) and 13 other variables. These variables were seasonal (four seasons) temperature in 2005, 2010, 2000, and mean annual temperature in 2000.  To clarify this point and to address the Editor’s concern, we added:   - a supplementary Table (S2 Table: Variables with significant correlation (p<0.05) with mean annual temperature in 2005 in Kyrgyzstan) showing the correlation coefficient of variables with significant correlation (p<0.05) with 10-year lagged temperature, as well as the sentences below in the results and discussion - the following sentence in the results section:   “Thirteen variables among the 190 variables considered initially were excluded from the BYM model for AE because they exhibited collinearity with the mean 10-year lag temperature (*i.e*., the significant predictor for AE). These variables were seasonal (four seasons) temperature in 2005, 2010, 2000, and mean annual temperature in 2000 (S2 Table).   - The following sentence in the discussion:   “Multicollinearity detected between the mean 10-year lag temperature and other mean temperature variables (S2 Table) can be expected when a variable computed in different time points displays limited temporal variability or strong spatial patterns. In our study, it is possible that the stability or strong spatial patterns of temperature in Kyrgyzstan might be due to the topography and landscapes (*e.g.*, rugged relief with valleys) that play an important role in defining climatic regions.” |
| C 2 | I see some discrepancy between reviewers' concerns that limitations were addressed (mainly the limitation of using surgical cases as a proxy for all cases). I agree with reviewer 3 that more explanation of how focusing on surgical cases might limit interpretations is warranted (rather than simply a statement that surgical cases limit interpretation - in what ways exactly would this bias the results)? | We appreciate this insightful comment.  We added the sentence below in the discussion to better describe in what way the use of surgical cases as a proxy for all cases could bias our results:  “This might bias our results by underestimating or overestimating the association between environmental and climatic variables and the number of disease cases. For example, the association between the mean 10-year lag temperature and the number of AE cases could be underestimated if the distribution of surgical cases and that of less advanced or AE cases would be clustered in the same way, or overestimated, should the less advanced or non-diagnosed AE cases be widespread across the country. Active surveillance (*e.g.*, ultrasound surverys) throughout Kyrgyzstan could provide data on the number and location of less advanced disease cases. However, such population-based imaging studies are usually done in limited areas of known high endemicity, such as the study in the village of Sary Mogol in southern Kyrgyzstan [39]. This village was identified as an AE cluster based on hospital records of AE surgical cases. The ultrasound survery performed in 2012 on 1617 persons confirmed the cluster uncovering a high prevalence of 4.2% probable or confirmed AE cases. A cross-sectional ultrasound-based survey based on a large sample size (over 24000 volunteers from 50 villages) was done in rural Bulgaria, Romania, and Turkey to estimate CE prevalence in Eastern Europe [4]. This study found active CE cysts in people of all ages and in all investigated villages. These villages were located in provinces with annual CE hospital incidence within the mid-range for the respective countries. Thus, we can assume that also in Kyrgyzstan the distribution of severe and less severe CE cases might be similar, minimizing possible biases.  Financial constraints preventing access to diagnosis and treatment might influence the number of diagnosed surgical cases and their location. However, previous analyses on the same data set showed no significant correlation between the location of the reported AE or CE surgical cases and the number or distance to the closest health facility in Kyrgyz local communities [8]. This provides evidence that possible bias is minimized. Finally, the size of our data set (2359 cases of CE and 546 cases of AE from a short time period of 3 years) adds robustness to our analyses, even if non-surgical cases could not be analyzed.” |
| **C 3** | Consider to add a lifecycle figure for each parasite, with the lifecycle figures enhanced to show where environmental variables are hypothesized to influence transmission. This basic mechanistic information about the potential direct and indirect influences of environmental variables on CE and AE transmission is hard to glean from the current manuscript as written. | We agree with the Editor that such figure would help understanding how climatic and environmental variables are hypothesized to influence transmission. We think the conceptual diagram of direct and indirect ecological influences on transmission of *Echinococcus granulosus* and *E. multilocularis* published by Atkinson and colleagues, 2013, provides a perfect depiction of this. Thus, we expanded (**bold words** below) the explanation on how ecological factors might impact parasites transmission and added the reference to the conceptual diagram of Atkinson and colleagues in the introduction:  “This is because environmental, topographic, and climatic risk factors, can affect the prevalence and geographical distribution of parasitic zoonoses such as CE and AE by influencing the survival and longevity of parasites’ eggs in the environment, **exposure to eggs,** presence and abundance of competent hosts, **their spatial overlap and predation** [9–12]. **Direct and indirect ecological influences on transmission of *E. granulosus* and *E. multilocularis* are depicted in the conceptual diagram of Atkinson *et al*.** [11].” |
| C 4 | Line 285 references a Table 5 but I do not see a table 5 in the manuscript - please rectify? | We thank the Editor for spotting this the typo. We corrected the typo (Table 5 -> Table 4). Additionally, we corrected another typo (Table 4 -> Table 3) in the previous paragraph. |
| C 5 | Finally, when you refer to relative risk throughout the manuscript, it is unclear relative to what? Please state your definition of relative risk explicitly. | We thank the editor for this important suggestion. The definition of relative risk is stated in the last paragraph of the methods, however, we agree it is important to add it also in the introduction of the manuscript to further clarify:  “[…] and mapped community-level relative risk for both diseases. Relative risk was defined as the ratio between fitted cases and expected cases at the community level.” |
|  | Editor comment  We cannot make any decision about publication until we have seen the revised manuscript and your response to the reviewers' comments. Your revised manuscript is also likely to be sent to reviewers for further evaluation.  When you are ready to resubmit, please upload the following:  [1] A letter containing a detailed list of your responses to the review comments and a description of the changes you have made in the manuscript. Please note while forming your response, if your article is accepted, you may have the opportunity to make the peer review history publicly available. The record will include editor decision letters (with reviews) and your responses to reviewer comments. If eligible, we will contact you to opt in or out.  [2] **Two versions of the revised manuscript**: one with either highlights or tracked changes denoting where the text has been changed; the other a clean version (uploaded as the manuscript file).   Important additional instructions are given below your reviewer comments.  Please prepare and submit your revised manuscript within 60 days. If you anticipate any delay, please let us know the expected resubmission date by replying to this email. Please note that revised manuscripts received after the 60-day due date may require evaluation and peer review similar to newly submitted manuscripts.  Thank you again for your submission. We hope that our editorial process has been constructive so far, and we welcome your feedback at any time. Please don't hesitate to contact us if you have any questions or comments.  Sincerely,  Susanne H. Sokolow  Associate Editor  PLOS Neglected Tropical Diseases  Justin Remais  Deputy Editor PLOS Neglected Tropical Diseases | |

# Reviewer's Responses to Questions

| Key Review Criteria Required for Acceptance?  As you describe the new analyses required for acceptance, please consider the following:  Methods  -Are the objectives of the study clearly articulated with a clear testable hypothesis stated?  -Is the study design appropriate to address the stated objectives?  -Is the population clearly described and appropriate for the hypothesis being tested?  -Is the sample size sufficient to ensure adequate power to address the hypothesis being tested?  -Were correct statistical analysis used to support conclusions?  -Are there concerns about ethical or regulatory requirements being met? | | |
| --- | --- | --- |
| **Comment #** | **Reviewer comment** | **Authors answer** |
| 1 | Reviewer #1: Yes | We thank Reviewer 1 for the positive feedback to points 1-6. |
| 2 | Reviewer #2:   1. Are the objectives of the study clearly articulated with a clear testable hypothesis stated?   Yes.   2. Is the study design appropriate to address the stated objectives?   Yes, with some limitations that, nevertheless, are clearly presented and discussed in the manuscript   3. Is the population clearly described and appropriate for the hypothesis being tested?   Yes.   4. Is the sample size sufficient to ensure adequate power to address the hypothesis being tested?   Unfortunately, Authors could only use surgery cases for AE and CE as response variable, instead of true notified cases (most likely not-reported/under-reported/misdiagnosed). However, I agree that these data were actually the best available option, so I won’t stress this point as a major flaw of their study at all.   5. Were correct statistical analysis used to support conclusions?   Yes.   6. Are there concerns about ethical or regulatory requirements being met?   No concerns. | 1. We thank Reviewer 2 for the positive feedback. 2. We thank Reviewer 2 for the positive feedback. 3. We thank Reviewer 2 for the positive feedback. 4. Thank you for highlighting this important study limitation, which was a concern for the Editor and for Reviewers 3 as well. The national CE and AE surveillance system in Kyrgyzstan is based on surgical cases, which are mandatorily reported to the health authorities as explained in lines 343-344. No data on alternative, less invasive treatment options is systematically collected (for example, by means of active surveillance). Thus, as the reviewer commented, these data were actually the only and best available option. Although we think the sample size of our data set (2359 cases of CE and 546 cases of AE from a short time period of 3 years) provides robustness to our analyses, we added the paragraph below in the discussion to further discuss possible biases due to this data limitation:   “This might bias our results by underestimating or overestimating the association between environmental and climatic variables and the number of disease cases. For example, the association between the mean 10-year lag temperature and the number of AE cases could be underestimated if the distribution of surgical cases and that of less advanced or AE cases would be clustered in the same way, or overestimated, should the less advanced or non-diagnosed AE cases be widespread across the country. Active surveillance (*e.g.*, ultrasound surverys) throughout Kyrgyzstan could provide data on the number and location of less advanced disease cases. However, such population-based imaging studies are usually done in limited areas of known high endemicity, such as the study in the village of Sary Mogol in southern Kyrgyzstan [39]. This village was identified as an AE cluster based on hospital records of AE surgical cases. The ultrasound survery performed in 2012 on 1617 persons confirmed the cluster uncovering a high prevalence of 4.2% probable or confirmed AE cases. A cross-sectional ultrasound-based survey based on a large sample size (over 24000 volunteers from 50 villages) was done in rural Bulgaria, Romania, and Turkey to estimate CE prevalence in Eastern Europe [4]. This study found active CE cysts in people of all ages and in all investigated villages. These villages were located in provinces with annual CE hospital incidence within the mid-range for the respective countries. Thus, we can assume that also in Kyrgyzstan the distribution of severe and less severe CE cases might be similar, minimizing possible biases.  Financial constraints preventing access to diagnosis and treatment might influence the number of diagnosed surgical cases and their location. However, previous analyses on the same data set showed no significant correlation between the location of the reported AE or CE surgical cases and the number or distance to the closest health facility in Kyrgyz local communities [8]. This provides evidence that possible bias is minimized. Finally, the size of our data set (2359 cases of CE and 546 cases of AE from a short time period of 3 years) adds robustness to our analyses, even if non-surgical cases could not be analyzed.”   5. We thank Reviewer 2 for the positive feedback. 6. We thank Reviewer 2 for the positive feedback. |
| 3 | Reviewer #3:   1. The objectives of the study should be more clearly stated in the last paragraph of the introduction. 2. As you are only studying surgical cases, which represent a small subset of total prevalent cases, the inherent bias in this study design should be clearly and extensively addressed in the discussion. 3. Statistical methodologies and modeling were sound and well described. No concerns with ethics. 4. Recommend disclosing the approved protocol number for transparency. 5. It would be nice to have a supplemental table with the numbers of surgical incidence cases (primary cases that were aggregated) by geographic area/unit. 6. It would improve understanding to have additional specific information about where the initial 190 geospatial variables were derived from (previous literature? other?). | 1. To better state the aims of our study, as per Reviewer 3 request, we rephrased the last sentence of the introduction (*i.e.*, “This explored potential associations between ecological determinants and the spatial patterns of CE or AE cases and identified high risk areas for designing appropriate public health interventions”) with the following sentence:   “Our modelling effort aimed at assessing potential associations between the spatial distribution of the CE and AE and ecological determinants, such as temperature or elevation. Our findings are meant to identify high risk areas to inform public health interventions.”   2. Thank you for highlighting this important limitation in our manuscript. The Editor and Reviewer 2 also raised this point. We therefore added the paragraph below in the discussion to better specify possible biases due to the use of surgical data:   “This might bias our results by underestimating or overestimating the association between environmental and climatic variables and the number of disease cases. For example, the association between the mean 10-year lag temperature and the number of AE cases could be underestimated if the distribution of surgical cases and that of less advanced or AE cases would be clustered in the same way, or overestimated, should the less advanced or non-diagnosed AE cases be widespread across the country. Active surveillance (*e.g.*, ultrasound surverys) throughout Kyrgyzstan could provide data on the number and location of less advanced disease cases. However, such population-based imaging studies are usually done in limited areas of known high endemicity, such as the study in the village of Sary Mogol in southern Kyrgyzstan [39]. This village was identified as an AE cluster based on hospital records of AE surgical cases. The ultrasound survery performed in 2012 on 1617 persons confirmed the cluster uncovering a high prevalence of 4.2% probable or confirmed AE cases. A cross-sectional ultrasound-based survey based on a large sample size (over 24000 volunteers from 50 villages) was done in rural Bulgaria, Romania, and Turkey to estimate CE prevalence in Eastern Europe [4]. This study found active CE cysts in people of all ages and in all investigated villages. These villages were located in provinces with annual CE hospital incidence within the mid-range for the respective countries. Thus, we can assume that also in Kyrgyzstan the distribution of severe and less severe CE cases might be similar, minimizing possible biases.  Financial constraints preventing access to diagnosis and treatment might influence the number of diagnosed surgical cases and their location. However, previous analyses on the same data set showed no significant correlation between the location of the reported AE or CE surgical cases and the number or distance to the closest health facility in Kyrgyz local communities [8]. This provides evidence that possible bias is minimized. Finally, the size of our data set (2359 cases of CE and 546 cases of AE from a short time period of 3 years) adds robustness to our analyses, even if non-surgical cases could not be analyzed.”   3. We thank Reviewer 3 for positive feedback. 4. We thank Reviewer 3 for the recommendation, however, the disclosure of an approved protocol number is not applicable in our study (retrospective analysis of national surveillance data) 5. We thank Reviewer 3 for the interest. To provide data on CE and AE cases aggregated at different spatial scales, we added the sentence below in the Methods section (3.1):   “Number of CE and AE cases aggregated at different spatial levels are available in the appendix of the work of Paternoster *et al*. [8].”   6. We thank the Reviewer for this comment. For clariry, we moved this important information (sentence in **bold** below) from lines 150-151 to the beginning of the methods section (3.2 Explanatory variables preprocessing and selection):   “**Based on previous ecological studies on AE and CE [9-11, 14, 21-28],**” we derived 190 geospatial variables on potential environmental and climatic risk factors for CE and AE from different satellite-based remote-sensing data sources. |
| Results  -Does the analysis presented match the analysis plan?  -Are the results clearly and completely presented?  -Are the figures (Tables, Images) of sufficient quality for clarity? | | |
| 4 | Reviewer #1:  Results are not well described. Part of the results section still look like methodology. Results are not completely presented. | We thank Reviewer 1 for this comment. We attempted to make the results section clearer with the corrections below. However, it is difficult to know how to proceed given that Reviewers 2 and 3 consider the results section clear, well presented, and with a high degree of detail.   - We removed one sentence which was redundant with the methods (i.e., lines 222-224: “For both diseases, we removed distance to settlements in 2005 and 2010 because redundant with distance to settlements in 2000.”) - We removed “Maps of estimated relative risk (RR) for CE (Fig 1) and AE (Fig 2) show distinctive spatial patterns.” Leaving only the spatial patterns description. - We added the following paragraph as well as a Supplementary table (S2 table):   “Thirteen variables among the 190 variables considered initially were excluded from the BYM model for AE because they exhibited collinearity with the mean 10-year lag temperature (*i.e.*, the significant predictor for AE). These variables were seasonal (four seasons) temperature in 2005, 2010, 2000, and mean annual temperature in 2000 (S2 Table).   - We emphasized that additional results (19 interactive maps and 19 tables) are available under this link https://www.math.uzh.ch/pages/kgz_eco/ in the results section |
| 5 | Reviewer #2:   1. -Does the analysis presented match the analysis plan?   Yes.   2. -Are the results clearly and completely presented?   Yes.   3. -Are the figures (Tables, Images) of sufficient quality for clarity?   Yes. Maybe a physical landscape map (graphical abstract?) could help the reader that is not familiar with Kyrgyzstan to understand the Country’s territory? | 1. We thank Reviewer 2 for the positive feedback. 2. We thank Reviewer 2 for the positive feedback. 3. Point 3: We wanted to avoid replication of the topography map we produced for our previous study, also considering the easy access to such maps. |
| 6 | Reviewer #3:   1. The analysis and results are clearly presented and match the methodology. 2. Tables are clear and concise. 3. Figures are nicely presented and clear. | We thank Reviewer 3 for the very positive feedback! |
| Conclusions  -Are the conclusions supported by the data presented?  -Are the limitations of analysis clearly described?  -Do the authors discuss how these data can be helpful to advance our understanding of the topic under study?  -Is public health relevance addressed? | | |
| 7 | Reviewer #1: Yes | We thank Reviewer 1 for the positive feedback on all points! |
| 8 | Reviewer #2:   1. Are the conclusions supported by the data presented? Yes. 2. Are the limitations of analysis clearly described? Absolutely. 3. Do the authors discuss how these data can be helpful to advance our understanding of the topic under study? Yes. 4. Is public health relevance addressed? Yes. | We thank Reviewer 2 for the very positive feedback on all points! |
| 9 | Reviewer #3:   1. Although the discussion of the explanatory variables is thorough, the limitations of the study group - surgical cases - need to be emphasized. Surgical incidence is not a strong proxy for transmission, and should be cautiously and thoughtfully used a proxy for disease incidence. Additional sentences in the discussion about potential implications of this limitation should be added. 2. Although you do touch on the utility of socioeconomic factors to be considered in disease distribution, it is worth adding that these factors influence healthcare access to reach diagnosis and/or surgery in cases.Thanks | We thank Reviewer 3 for the insightful comments.  We addressed points 1 and 2 by adding the following sentence in the discussion:  “This might bias our results by underestimating or overestimating the association between environmental and climatic variables and the number of disease cases. For example, the association between the mean 10-year lag temperature and the number of AE cases could be underestimated if the distribution of surgical cases and that of less advanced or AE cases would be clustered in the same way, or overestimated, should the less advanced or non-diagnosed AE cases be widespread across the country. Active surveillance (*e.g.*, ultrasound surverys) throughout Kyrgyzstan could provide data on the number and location of less advanced disease cases. However, such population-based imaging studies are usually done in limited areas of known high endemicity, such as the study in the village of Sary Mogol in southern Kyrgyzstan [39]. This village was identified as an AE cluster based on hospital records of AE surgical cases. The ultrasound survery performed in 2012 on 1617 persons confirmed the cluster uncovering a high prevalence of 4.2% probable or confirmed AE cases. A cross-sectional ultrasound-based survey based on a large sample size (over 24000 volunteers from 50 villages) was done in rural Bulgaria, Romania, and Turkey to estimate CE prevalence in Eastern Europe [4]. This study found active CE cysts in people of all ages and in all investigated villages. These villages were located in provinces with annual CE hospital incidence within the mid-range for the respective countries. Thus, we can assume that also in Kyrgyzstan the distribution of severe and less severe CE cases might be similar, minimizing possible biases.  Financial constraints preventing access to diagnosis and treatment might influence the number of diagnosed surgical cases and their location. However, previous analyses on the same data set showed no significant correlation between the location of the reported AE or CE surgical cases and the number or distance to the closest health facility in Kyrgyz local communities [8]. This provides evidence that possible bias is minimized. Finally, the size of our data set (2359 cases of CE and 546 cases of AE from a short time period of 3 years) adds robustness to our analyses, even if non-surgical cases could not be analyzed.” |
| Editorial and Data Presentation Modifications?  Use this section for **editorial suggestions** as well as **relatively minor modifications of existing data that would enhance clarity**. If the only modifications needed are minor and/or editorial, you may wish to recommend “Minor Revision” or “Accept”. | | |
|  | Reviewer #1: Not applicable | We thank Reviewer 1 for the positive feedback. |
|  | Reviewer #2: |  |
| 10 | ABSTRACT  - Line 36: I disagree with AE and CE being called “emerging” zoonoses. Is that the case for Kyrgyzstan? Or is it rather an endemic region? please clarify your statement or either just rename them properly as “zoonotic parasitic infections” or “neglected tropical diseases”  - Line 46: 10-year lag? | - Line 36: we thank Reviewer 2 for this comment. There is evidence that *E. multilocularis* has always been present in the wild life (described in Soviet literature from the 1950s), but only recently have large numbers of human AE cases been reported in Kyrgyzstan (<https://www.ncbi.nlm.nih.gov/pmc/articles/PMC6004877/>, <https://pubmed.ncbi.nlm.nih.gov/23763935/>). Thus, it is certainly an emerging human disease in this country. CE likewise is reemerging (<https://www.thelancet.com/journals/langlo/article/PIIS2214-109X(20)30038-3/fulltext)>. Furthermore, AE seems to be an emerging public health threat in Europe (<https://www.cell.com/trends/parasitology/pdf/S1471-4922(15)00118-X.pdf>) and potentially in North America (<https://pubmed.ncbi.nlm.nih.gov/25531581/>). CE or AE are referred as “emerging” or “re-emerging in many regions of the world, especially in regions of North America, Europe and Asia: - <https://pubmed.ncbi.nlm.nih.gov/30276210/> - <https://pubmed.ncbi.nlm.nih.gov/26895758/> - <https://pubmed.ncbi.nlm.nih.gov/29975650/> - <https://pubmed.ncbi.nlm.nih.gov/32095619/> - <https://pubmed.ncbi.nlm.nih.gov/31242932/> - <https://pubmed.ncbi.nlm.nih.gov/31193569/> - <https://pubmed.ncbi.nlm.nih.gov/32448547/> - <https://pubmed.ncbi.nlm.nih.gov/33220298/> - <https://pubmed.ncbi.nlm.nih.gov/27178627/> - <https://pubmed.ncbi.nlm.nih.gov/28270242/>   As per Reviewer 2 suggestion, we renamed the diseases as well as clarified the abstract **(bold** words) as follows:  “Cystic and alveolar echinococcosis (CE and AE) are **neglected tropical diseases** caused by *Echinococcus granulosus sensu lato* and *E. multilocularis****,*** **and are emerging zoonoses in Kyrgyzstan**. **In this country,** the spatial distribution […]”   - Line 46: to define variables calculated 10 years before a certain event (in this case, the study period) expressions such as “*variable* at (a) *x*-year lag” or “*x*-year lag *variable*” seems to be accepted, as in the work of Cadavid Restrepo and colleagues: <https://parasitesandvectors.biomedcentral.com/articles/10.1186/s13071-018-2693-z>. |
| 11 | INTRODUCTION  - LINE 74: same as for line 36. Pls check all through the manuscript  - Line 95: is there some infection prevalence data for your Country / neighbouring areas?  - Line 116: spatial variation OF AE | - Line 74: We thank Reviewer 2 for the comment. Considering the references mentioned above for the comment on LINE 36, we edited (**bold** words below) the term “emerging disease/s” throughout the manuscript as follows:   - Line 74: “ add to the body of knowledge on the ecology of these neglected parasitic diseases that are emerging and reemerging **~~globally~~** **in several regions in North America, Europe and Asia**.   - Line 78: “Cystic echinococcosis (CE) and alveolar echinococcosis (AE) are **~~emerging~~** parasitic zoonoses caused by Echinococcus granulosus sensu lato and E. multilocularis, respectively. - Line 95: we added the following paragraph (**in bold**) to add information on CE and AE in Central Asia:   “Kyrgyzstan is a mountainous landlocked **country located in Central Asia, bordered by Kazakhstan, Uzbekistan, Tajikistan and China. The country has a** continental and mostly arid climate [6] covering an area of 199,951 km2. Kyrgyzstan is divided into 7 regions. Bishkek, the capital, and the city of Osh and are administratively equivalent to regions. Further administrative divisions are 56 districts and 479 local communities. The 2016 estimated country’s population was ≈ 6 million people [7]. **In Central Asia, including Kyrgyzstan and its neighbouring countries, both CE and AE appear to be emerging, particularly in the last 30 years after the dissolution of the Soviet Union in 1991. Over 90% of the global burden of AE occurs in neighboring China with over 16000 annual reported AE cases** [2].   - Line 116: amended as suggested. |
| 12 | M&M   - Line 132: paragraph “3.1 Incidence data” mostly describes what authors did in a previous publication (reference 8). Please consider to shorten this part, since methods were fully described elsewhere. - Line 139: please clarify “independent cities - Line 213: please check here (www.r-bloggers.com/2018/06/its-easy-to-cite-and-reference-r/) or here (<https://cran.r-project.org/doc/FAQ/R-FAQ.html>) how to correctly cite R software and its packages. It should go as: (R Core Team, YEAR), with YEAR = release year of the version of R you used. | - Line 132: we thank Reviewer 2 for this suggestion. We shortened the paragraph “3.1 Incidence data” by removing 59 words obtaining the following paragraph:   “As fully described in Paternoster *et al.* [8], we obtained records of confirmed CE and AE surgical cases in Kyrgyzstan between 2014 and 2016 from the Government Sanito-Epidemiology Unit in Bishkek. Each case was manually geocoded according to the patient’s community residence, which was assumed to be a proxy for the place of infection. Primary cases (i.e., excluding relapses) were then aggregated at the community-level (n=490) and linked to the 2009 national census population data to estimate average annual crude surgical incidence of CE and AE at the community level in 2014-2016 [8]. We also computed the number of expected cases of CE and AE using indirect standardization, and standardized incidence ratios (SIR) for both diseases at the community level [8].   - Line 139: “independent city” was removed following the shortening of the paragraph (above). These cities are a peculiarity of the Kyrgyz administrative system. The administrative level 2 includes 56 units, of which 44 are district and 12 are “independent cities” with such numerous population to be considered as districts. These peculiarities are described in reference 8 and its appendix. - We thank Reviewer 2 for this suggestion. We cited R software as follows:   R Core Team (2018). R: A language and environment for statistical computing. R Foundation for Statistical Computing, Vienna, Austria. URL https://www.R-project.org/. |
| 13 | RESULTS   1. Lines 222-224: “For both diseases, we removed distance to settlements in 2005 and 2010 because redundant with distance to settlements in 2000.” Authors already stated this in M&M. please delete from here to avoid redundancy. 2. Line 226: please rephrase as “For each selected explanatory variable, a descriptive report including (…)” 3. Table 1: First column – maybe “response variable” selected for modelling fits better..? 4. Line 239: “12 did not have neighbors” please provide better explanation 5. Line 242: I think “The higher DIC values for the IND models indicate that for both diseases, the model fit was improved by additional spatial random effect” is somehow clearer 6. Lines 285 & 290: I couldn’t open the url | 1. Lines 222-224: we thank Reviewer 2 for spotting this redundancy. We amended the text as suggested. 2. Line 226: thank you, we rephrased the sentence as suggested and it reads much better. 3. Table 1, first column: we renamed “response variable” selected for modelling as suggested as we agree that it fits better. Thank you. 4. Line 239: thank you for this comment. We attempted to add clarity by expanding (**bold words**) the sentence as follows:   “12 did not have **neighboring communities** [8] and were thus excluded (…). **Kyrgyz communities have the peculiarity of not covering the entire country, but only the settlements and their surroundings. Twelve communities in this country do not border on other communities.”**   5. Line 242: thank you, we rephrased as suggested to add clarity. 6. Lines 285 & 290: thank you very much, we replaced the url with “Map source: supplementary appendix of [8], pp 6-9.” |
| 14 | DISCUSSION  Line 327: Similarly | DISCUSSION  Line 327: amended as suggested, thank you. |
|  | Reviewer #3: See editorial comments by line below: |  |
| 15 | Line 87 - this sentence is unclear; consider rewording to "....AE) metacestode development differs for CE and AE" or similar | Line 87: amended as suggested, thank you. |
| 16 | Line 88 - recommend changing "fluid-filled metacestodes" to hydatid cysts | Line 88: we added “called hydatid cysts” for clarity. Thank you. |
| 17 | Line 89,90 - AE lesions are really infiltrative proliferations of metcestode cells....consider rewording protrusions | Line 89,90:thak you for the comment, we expanded (**bold words**) the sentence as follows: “infiltrat**ing cellular** protrusions of the **metcestode’s germinal layer”** |
| 18 | Line 94 - change "landlock" to "landlocked"; "sharp continental" unclear intent in this wording | Line 94: thank you, we changed change "landlock" to "landlocked” and "sharp continental" to "continental”. |
| 19 | Line 97 to 98- notifiable to what agency? | Line 97-98: thank you, we added “to the to the Government Sanito-Epidemiology Unit in Bishkek” |
| 20 | Lines 110 - 115 may be removed beginning at paragraph start and ending at "hypotheses in disease transmission dynamics" | Lines 110 – 115: we thank Reviewer 3 for this comment. However, we think it is important to leave this paragraph on spatial epidemiology methods. We think these methods are worth to be mentioned with the relative references and shared for further applications in the NTDs environmental modelling field, as mentioned by Reviewer 2 in the final general comments. |
| 21 | Line 130 - provide approved protocol number if available | We thank Reviewer 3 for the recommendation; however, an approved protocol number is not available for our study (retrospective analysis of national surveillance data). |
| 22 | Line 133 - use authors name et al. in "As described in..." | Line 133: thank you, amended as suggested. |
| 23 | Line 136 - replace "surrogate" with "proxy" (also, not that place of residence may not likely represent place of infection, especially in communities where farmers travel for work, etc.) | Line 136: we replaced "surrogate" with "proxy" as suggested. The majority of the residence communities we used as proxy for the place of infection has an area up to 150 sqKm, which includes pasture and surroundings of villages where farmers travel for work. |
| 24 | Line 293 - remove "potential"; the word potential is used twice in that sentence | Line 293: amended as suggested, thank you. |
| 25 | Line 345 - change to "are often misdiagnosed and underreported" | Line 345: Thank you, we changed to "are often misdiagnosed and underreported" as suggested. |
| 26 | Line 349 - change "infectious moment" to "moment of infection" | Line 349: we thank Reviewer 32 for this suggestion. We changes as suggested and we think it reads better. |
| 27 | Line 357 - remove hyphen from socio-economic (socioeconomic) | Line 357: we removed the hyphen from socio-economic as recommended. Thank you. |
| 28 | Lines 367 to 371 - reword these for clarity and eloquence | Lines 367-371: we thank Reviewer 3 for this comment. To improve clarity and eloquence we rephrased as follows:  “To identify socioeconomic and behavioral risk factors for CE and AE in Kyrgyzstan additional population-based studies are needed. Nevertheless, this study indicates that temperature plays an important explanatory role in determining the AE spatial distribution. Therefore climate change may affect this distribution and spatial modelling could be used to predict where the risk of occurrence will be in response to future rising temperatures.” |
| Summary and General Comments  Use this section to provide overall comments, discuss strengths/weaknesses of the study, novelty, significance, general execution and scholarship. You may also include additional comments for the author, including concerns about dual publication, research ethics, or publication ethics. If requesting major revision, please articulate the new experiments that are needed. | | |
|  | Reviewer #1: | |
| 29 | 1. This is a great research describing potential association between environmental/climatic risk factors and the spatial distribution of cystic and alveolar echinococcosis. 2. It can be further revised to generally address some long winding (and perhaps unnecessary) statements. 3. Results are not well described. Any part of the results that has to do with 'how the work was performed' should be taken to Methods. Likewise, any part of the results that is describing implication of the result should be transferred to Discussion. Please state or describe your results in detail under Results. 4. Give implications of the study or results in Discussion section. 5. Please minimize the use of the word "we", it makes the paper boring - you may rather use third person reporting format in most cases. 6. In line 95, did you mean Kyrgyzstan is divided in 7 regions, 2 main cities ... or into 7 regions, ...? | 1. We thank Reviewer 1 for the very positive feedback as well as for all the inputs and suggestions that improved both manuscript quality and readability. 2. We thank Reviewer 1 for this comment. To improve clarity and conciseness we shortened the methods section and rephrased the last paragraph of the discussion as well as amending the text according to the suggestions of the Editors and the Reviewers. 3. We thank Reviewer 1 for this comment. We attempted to make the results section clearer with the corrections below. However, it is difficult to know how to proceed given that Reviewers 2 and 3 consider the results section clear, well presented, and with a high degree of detail.    - We removed one sentence which was redundant with the methods (i.e., lines 222-224: “For both diseases, we removed distance to settlements in 2005 and 2010 because redundant with distance to settlements in 2000.”)    - We removed “Maps of estimated relative risk (RR) for CE (Fig 1) and AE (Fig 2) show distinctive spatial patterns.” Leaving only the spatial patterns description.    - We added the following paragraph as well as a Supplementary table:   “Thirteen variables among the 190 variables considered initially were excluded from the BYM model for AE because they exhibited collinearity with the mean 10-year lag temperature (i.e., the significant predictor for AE). These variables were seasonal (four seasons) temperature in 2005, 2010, 2000, and mean annual temperature in 2000 (S2Table).   - We emphasized that additional results (19 interactive maps and 19 tables) are available under this link https://www.math.uzh.ch/pages/kgz_eco/ in the results section  4. We thank Reviewer 1 for this suggestion, we attempted to better rephrase the last paragraph of the discussion to clarify the implications of the study:   “Our spatial analyses of ecologic determinants of CE and AE distribution allowed to produce relative risk maps that can help targeting public health interventions in communities where CE and AE might be under- or misdiagnosed. These communities could be targeted by area-specific health education (e.g., early disease detection and treatment, prevention in the animal hosts, information campaigns for the general population) and active surveillance (e.g. ultrasound survey in the population). To identify socioeconomic and behavioural risk factors for CE and AE in Kyrgyzstan additional population-based studies are needed. Nevertheless, this study indicates that temperature plays an important explanatory role in determining the AE spatial distribution. Therefore climate change may affect this distribution and spatial modelling could be used to predict where the risk of occurrence will be in response to future rising temperatures.”   5. We thank Reviewer 1 for this suggestion. Although the active form is usually recommended for scientific writing, we rephrased the sentences below into the passive form to improve the readability of the manuscript:    - “Relative risk was defined as the ratio between fitted cases”    - “Each case was manually geocoded according to the patient’s community residence, which was assumed to be a proxy for the place of infection.    - “Primary cases (i.e., excluding relapses) were then aggregated at the community-level (n=490) and linked to the 2009 national census population data”    - “, communities at risk where these diseases are potentially under- or misdiagnosed were identified.”    - “To account for the prolonged latency of CE (months to years) and AE (10–15 years) [5], variables were derived for the years 2000, 2005, and 2010”    - “Nonlinear correlations were also checked by visually inspecting pairwise scatterplots”    - “Variance inflation factor (VIF) was used to detect multicollinearity,”    - “Other variables were also included based on existing literature”    - “For all spatial models, CE and AE community-level cases counts were used as the response variable”    - “, communities with no neighbors were excluded from the analysis.”    - “model fit was improved by additional spatial random effect.” 6. Thank you for this comment. We expanded the sentence to clarify as follows:   “Kyrgyzstan is divided into 7 regions. Bishkek, the capital, and the city of Osh are administratively equivalent to regions. Further administrative divisions are 56 districts and 479 local communities.” |
|  | Reviewer #2: | |
| 30 | 1. The manuscript is generally well written and interesting. A high degree of detail was provided for Methods and also the study’s current main limitations (e.g. AE and CE surgery cases as response variable) were clearly presented and discussed. I really appreciated that. 2. The study might suffer from a limited geographical significance but, nevertheless, spatial epidemiology methods are certainly worth to be published & shared for further applications in the NTDs environmental modelling field. 3. I feel like the manuscript is actually suitable for publication thank to its M&M, after minor revision, on the one condition that the Editor finds their country-level “application” suitable for high-ranking PLOS-ntd. | 1. We thank Reviewer 2 for the interest and positive feedback on our study. 2. This countrywide study on two different diseases provides important epidemiological data. Large regions of western China, which borders Kyrgyzstan, are endemic for AE. Moreover, both CE and AE are considered to be re-emerging diseases in central Asia, with an estimated 270 million people are estimated to be at risk (<https://www.thelancet.com/pdfs/journals/langlo/PIIS2214-109X(20)30066-8.pdf>). 3. We thank Reviewer 2 again for the positive feedback on our study. |
|  | Reviewer #3: | |
| 31 | 1. This paper presents sound and interesting methodologies and the data is well presented; 2. however, there is concern for interpretation of the findings due to only the inclusion of surgical cases of CE/AE. The implications of using only surgical cases and the limitations of any conclusions in the study should be clearly detailed in the discussion. 3. Overall a very interesting read and clearly thorough background research was done in preparing these models. | 1. We thank Reviewer 3 for the positive feedback on our study. 2. We thank Reviewer 3 for the constructive comments and suggestions. We added a paragraph in the discussion to detail the implication and possible biases due to the use of surgical cases of CE and AE. 3. We thank Reviewer 3 again for the positive feedback and appreciation of our work. |

| Figure Files |  |
| --- | --- |
| While revising your submission, please upload your figure files to the Preflight Analysis and Conversion Engine (PACE) digital diagnostic tool, https://pacev2.apexcovantage.com. PACE helps ensure that figures meet PLOS requirements. To use PACE, you must first register as a user. Then, login and navigate to the UPLOAD tab, where you will find detailed instructions on how to use the tool. If you encounter any issues or have any questions when using PACE, please email us at figures@plos.org. |  |
| Data Requirements |  |
| Please note that, as a condition of publication, PLOS' data policy requires that you make available all data used to draw the conclusions outlined in your manuscript. Data must be deposited in an appropriate repository, included within the body of the manuscript, or uploaded as supporting information. This includes all numerical values that were used to generate graphs, histograms etc.. For an example see here: <http://www.plosbiology.org/article/info%3Adoi%2F10.1371%2Fjournal.pbio.1001908#s5>. |  |
