# Supplementary material for: Association between environmental and climatic risk factors and the spatial distribution of cystic and alveolar echinococcosis in Kyrgyzstan
Source: PLoS Negl Trop Dis. 2021 Jun 23;15(6):e0009498. doi: 10.1371/journal.pntd.0009498 (PMC8259979; doi:10.1371/journal.pntd.0009498)
Supplement: Attachment — Submitted filename: response to reveiwers.docx [file pntd.0009498.s008.docx]

Response to reviewers’ and editor’s comments.

In respect to the concern that the 10-year lag has a very definitive meaning rather than a 5 year or 15 year lag we have amended the abstract and the discussion as detailed below (addition in blue). As shown in the supplementary table, the correlations between the temperatures of different years is extremely high and this is now more specifically referenced in the discussion.

We have also removed the definition of multicollinearity as suggested by reviewer #3

Abstract

The number of AE cases was negatively associated with 10-year lag mean annual temperature. Although this time lag should not be considered as an exact measurement but with associated uncertainty, it is consistent with the estimated 10–15-year latency following AE infection.

Discussion

However, the moment of infection with *Echinococcus spp.* is not traceable in space and time [4]. Moreover, the correlation of temperature data from different years (S2 Table) shows a stability of this variable over time and space. Thus, the exact time lag between the measured temperature data and the associated higher number of cases cannot be precisely established from this dataset and should be considered with a degree of uncertainty.
